# Progress and Prospects of Microplastic Biodegradation Processes and Mechanisms: A Bibliometric Analysis

**DOI:** 10.3390/toxics12070463

**Published:** 2024-06-26

**Authors:** Yingnan Cao, Jing Bian, Yunping Han, Jianguo Liu, Yuping Ma, Weiying Feng, Yuxin Deng, Yaojiang Yu

**Affiliations:** 1 Key Laboratory of Environmental Pollution Control and Remediation at Universities of Inner Mongolia Autonomous Region, College of Resources and Environmental Engineering, Inner Mongolia University of Technology, Hohhot 010051, China; caoyingnan@imut.edu.cn (Y.C.); 17304630142@163.com (J.B.); waipomyp99@163.com (Y.M.); 18347429190@163.com (Y.Y.); 2 State Key Laboratory of Environmental Aquatic Chemistry, Research Center for Eco-Environmental Sciences, Chinese Academy of Sciences, Beijing 100085, China; 3 University of Chinese Academy of Sciences, Beijing 100049, China; 4 School of Materials Science and Engineering, Beihang University, Beijing 100191, China; fengweiying@buaa.edu.cn (W.F.); dengyx@buaa.edu.cn (Y.D.)

**Keywords:** microplastic, biodegradation, bibliometric, mechanism research

## Abstract

In order to visualize the content and development patterns of microplastic biodegradation research, the American Chemical Society (ACS), Elsevier, Springer Link, and American Society for Microbiology (ASM) were searched for the years 2012–2022 using Citespace and VOSvivewer for bibliometrics and visual analysis. The biodegradation processes and mechanisms of microplastics were reviewed on this basis. The results showed a sharp increase in the number of publications between 2012 and 2022, peaking in 2020–2021, with 62 more publications than the previous decade. The University of Chinese Academy of Sciences (UCAS), Northwest A&F University (NWAFU), and Chinese Academy of Agricultural Sciences (CAAS) are the top three research institutions in this field. Researchers are mainly located in China, The United States of America (USA), and India. Furthermore, the research in this field is primarily concerned with the screening of functional microorganisms, the determination of functional enzymes, and the analysis of microplastic biodegradation processes and mechanisms. These studies have revealed that the existing functional microorganisms for microplastic biodegradation are bacteria, predominantly Proteobacteria and Firmicutes; fungi, mainly Ascomycota; and some intestinal microorganisms. The main enzymes secreted in the process are hydrolase, oxidative, and depolymerization enzymes. Microorganisms degrade microplastics through the processes of colonization, biofilm retention, and bioenzymatic degradation. These studies have elucidated the current status of and problems in the microbial degradation of microplastics, and provide a direction for further research on the degradation process and molecular mechanism of functional microorganisms.

## 1. Introduction

“Microplastics” is used to describe plastic fibers, particles, and films that are less than 5 mm in size [1,2]. They are found in the air [3,4], oceans [5,6,7,8,9], soils [10,11], sediments, and surface waters [12,13,14,15,16,17] around the world, and have even been found in the sparsely populated Antarctic [18] and Arctic [19]. Some studies reported that the concentration of microplastics in surface water is between 10^−5^ and 10^5^ sheets/m^3^ and up to 67,500 mg/kg in soil [20,21,22,23,24]. Although there has been a great deal of research into their detection in the environment, there are difficulties in quantifying microplastics and nanoplastics, such as microplastics being too small to be fully captured and a lack of consistent standards for detection in different environments. In addition, a large number of microplastics in the environment are prone to migrate and transform in different environmental media due to their small particle size and low density, and release harmful substances such as phthalates (PAEs), polybrominated diphenyl ethers (PBDEs), and biphenyl A (BPA) in the process of migration and transformation [25]. Microplastics may also be compounded with persistent organic pollutants (POPs) and antibiotics to form composite pollutants in the ecosystem, thus causing harm to plants, animals, and microorganisms, and migrating into humans through the nutrient chain [26].

Due to the widespread presence of microplastics and their hazards, their removal from the environment has received considerable attention in recent years. The main removal methods include physical, chemical, and biological methods. The term “biological methods” refers to the use of microorganisms present in nature to remove microplastics from the environment. This is achieved through their metabolic activities, which further break up and split microplastics, decompose them into CO_2_ and H_2_O, and achieve inorganic mineralization. Biological degradation has been shown to be more effective than other methods, improving safety and environmental protection and contributing to the self-purification capacity of the contaminated environment. In terms of microplastic types, biodegradation focuses on both biodegradable and non-biodegradable microplastics. Biodegradable microplastics are environmentally friendly and can be completely degraded by microorganisms, while non-biodegradable microplastics degrade at a slower rate, with large differences in degradation efficiency and sometimes incomplete degradation. Common biodegradable microplastics are polylactic acid (PLA) and polybutylene terephthalate (PBAT). They are chemically bound to react readily in soil, water, and other environments, with microorganisms producing monomers through metabolic reactions. Common microorganisms include *Bacillaceae*, *Micromonosporaceae*, *Pseudonocardiaceae*, *Streptosporangiaceae*, and *Thermoactinomycetaceae*. In addition, under industrial composting conditions at 40 degrees Celsius, 80% of modified PLA fiber microplastics are completely degradable within a week, and Polycaprolactone (PCL) can be degraded in two days [27]. Recent studies have demonstrated that the metabolic processes of bacteria, fungi, and intestinal microorganisms facilitate the degradation of microplastics, rendering them a potential solution and effective mitigation measure for microplastic pollution [20,28]. *Exiguobacterium* sp. YT2 isolated from Tenebrio molitor larvae by Yang et al. formed biofilm on polystyrene (PS) after 28 days and reduced the weight by 7.4% in 60 days [29]. Similarly, Devi et al. isolated *Bacillus parapsilosis* (BP) and *Bacillus cereus* (BC) from the Vaigai River, India, and reduced the weight of Polypropylene (PP) and polyethylene (PE) by 78.99% and 63%, respectively [30]. Paco et al. found that the marine fungus *Zalerion maritimum* could use, and thus reduce the size and mass of, PE. The removal rate was as high as 43% after a 14-day degradation process [31].

In summary, current research has identified a large number of functional microorganisms and enzymes and explored the changes that occur in microplastics throughout time. However, there is still a lack of insight into the molecular mechanisms of biodegradation, the location of degradation, and the risk of degradation products. There is a need to comprehensively summarize current research, further explore the current knowledge on biodegradation by comparing the degradation of microplastics by different microorganisms, and study and understand the potential mechanisms of microplastic biodegradation in ecosystems in order to develop effective methods of microplastic removal and lay the foundation for future research.

Therefore, this review presents the first comprehensive analysis of microplastic biodegradation and its mechanisms using a bibliometric approach. Current research on microplastic biodegradation and its mechanisms was extracted from the published literature. Based on the number of studies available, their co-citation frequency, and keywords, the research hotspots of microplastic biodegradation and future research trends based on keyword clustering were derived to provide references for microplastic biodegradation research.

## 2. Materials and Methods

### 2.1. Data Retrieval

With “biodegradation of microplastics”, “microplastic biodegradation process”, and “microplastic biodegradation mechanism” as the keywords, four databases were comprehensively searched from inception to 2022: American Chemical Society (ACS), Elsevier, Springer Link, and American Society for Microbiology (ASM). No restrictions were implemented on the language, document type, data category, or year of publication. The data obtained from the four databases were consolidated, and a preliminary selection of identified publications was then undertaken. Since this study focuses on microplastics that are formed from large plastic particles through weathering and abrasion and exist in the environment for a long period of time, it excluded records of biodegradable microplastics produced by plastic products whose biodegradability was taken into account during the manufacturing process and studies focusing on microplastic–microorganism interactions and their impact on the ecosystem. For the articles that could not be identified, they were further filtered by reading the content.

### 2.2. Analysis Method

By means of bibliometric measures, we measured and visually analyzed the identified papers in the field of microplastic biodegradation, including the number of citations, top authors, active countries, institutions, and keywords. The articles were counted in Excel and categorized and mapped by type. A co-occurrence analysis of issuing countries, institutions, authors, and keywords was performed in CiteSpace (version 5.6. R 3) to count the number of articles published by different countries, institutions, and authors; to show the connection between them; and to show the hotspots of research in recent years in the field through co-occurring keywords and emergent. We used a table in VOSviewer (version 1.6.15) to count the co-cited journals and visualize the co-cited references to highlight the key contributions in the field.

## 3. Results

### 3.1. Paper Type and Quantity

**Figure 1:**
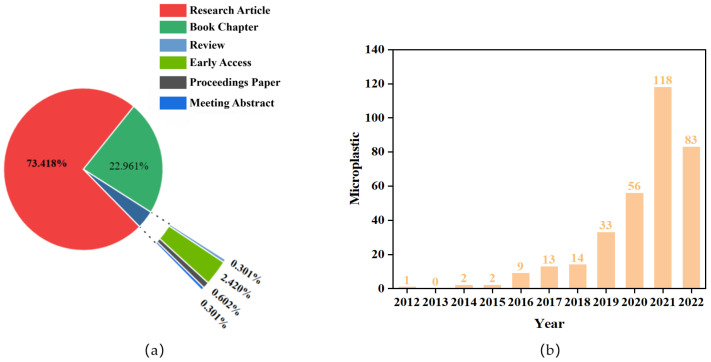
Paper type and quantity: (**a**) percentages of the types of the research records on microplastic biological management and mechanisms of microplastics; (**b**) number of studies published from 2012 to 2022 on the biological management and mechanisms of microplastics.

A total of 545 papers on microplastic biodegradation and its mechanisms were identified and examined, and 331 were retained after excluding irrelevant and redundant records. First, the statistics of the included studies were ranked and analyzed to reach preliminary conclusions. There were six types of publications in the search results (Figure 1a). Most of these were research articles (73.19%), followed by review papers (22.83%). Research articles and comments may reflect the most recent trends and changes in research on the biological governance and mechanisms of microplastics.

By examining the relationship between the timing and the number of publications, it is possible to reveal the past status of the research field and predict its future direction [32]. This strategy was used to improve the understanding of microplastic biodegradation and its mechanisms. As shown in Figure 1b, the research phase can be divided into two based on the characteristics of the variation in the annual volume of studies on microplastics: in the first stage, the increase in publications with time from 2012 to 2018 was stable, and the annual increase was low for the slow development stage; in the second stage, the number of publications increased rapidly from 2018 to 2022. By 2021, the number of global research articles on the biological governance and mechanisms of microplastics exceeded 100 for the first time, reaching 127, doubling the number of publications from the previous year. This may be related to the fact that microplastics were identified as a new pollutant in 2019 and their presence has been found in the human body [33].

### 3.2. Authors, Countries, and Institutions

To some extent, the number of publications published in specific research areas can reflect the impact on their field [34]. The top ten authors and co-cited authors were studied (Table 1). Currently, most authors in the field of microplastic biodegradation and mechanism research are from China, among whom Yong Zhang has the most papers.

**Figure 2:**
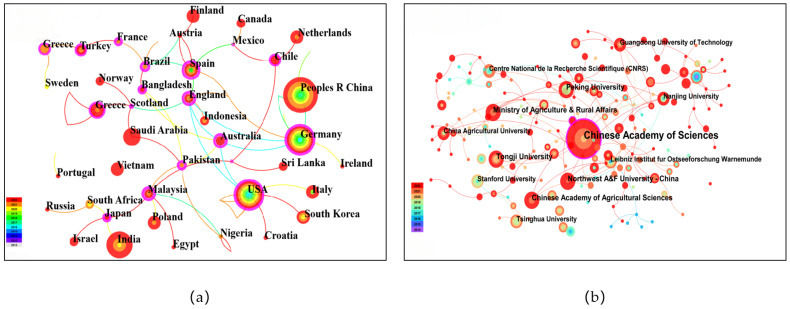
Country and institution network maps: (**a**) the top 20 productive countries; (**b**) the institutions conducting research on microplastic biological management and mechanisms of microplastics.

According to the analysis conducted using Citespace software, 81 countries and regions have published research papers on the biological governance and mechanisms of microplastics, among which the countries that have published within the top 10 countries are China, the United States of America, India, Italy, Germany, South Korea, England, Spain, Australia, and Greece. The top twenty countries in terms of publication number were studied, as were the links to the articles from each country (Figure 2a). The size of nodes symbolizes the number of papers; therefore, nodes become larger as the number of publications increases [35]. As can be seen from Figure 2a, the collaboration among countries is very close; China has the largest circle, representing the highest number of publications in the world. China’s close collaborating countries are mainly the United States of America, the United Kingdom, Germany, and Australia.

At present, 308 institutions have studied the biodegradation and mechanisms of microplastics. The Chinese Academy of Sciences represented the highest proportion at 8%, publishing 120 related papers, which accounted for 8%. The Chinese Academy of Agricultural Sciences came in second (5.8%) and Northwest A&F University came third (3.3%). Close cooperation was established between the institutions in each country. Among them, Nanjing University, the Chinese Academy of Sciences, and the University of the Chinese Academy of Sciences are connected with thick lines, indicating a close relationship (Figure 2b).

### 3.3. Co-Cited Journals and References

As shown in Table 2, Environmental Science & Technology is at the top of the list with 280 citations. Science of the Total Environment magazine ranks second and was cited 267 times. Of the five journals, the most cited number was concentrated in 2012.

**Figure 3:**
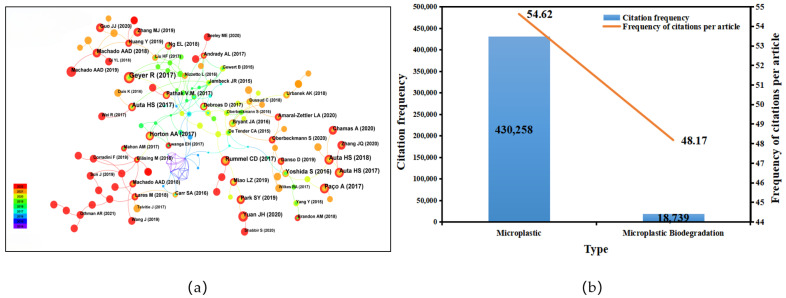
Co-reference literature analysis: (**a**) analysis of microplastic co-references; (**b**) frequency of the cited literature.

As shown in Figure 3a, Geyer et al. (2017) published a paper on microplastics in the marine environment with the most citations at 87. There were five papers with more than six citations, published by Rummel et al. (2017), Auta et al. (2018), Paco et al. (2017), and Yuan et al. (2020). Compared with the citation frequency of microplastic-related papers published in the same period, the citation frequency of microplastic biodegradation is slightly lower, which may be related to the late start of research in this area. However, the citation frequency of each article on microplastic biodegradation was the highest at 48.17, indicating that the academic value of each article is high and the results are gradually receiving more attention (Figure 3b).

### 3.4. Co-Occurrence and Burst Keywords Discussion

**Figure 4:**
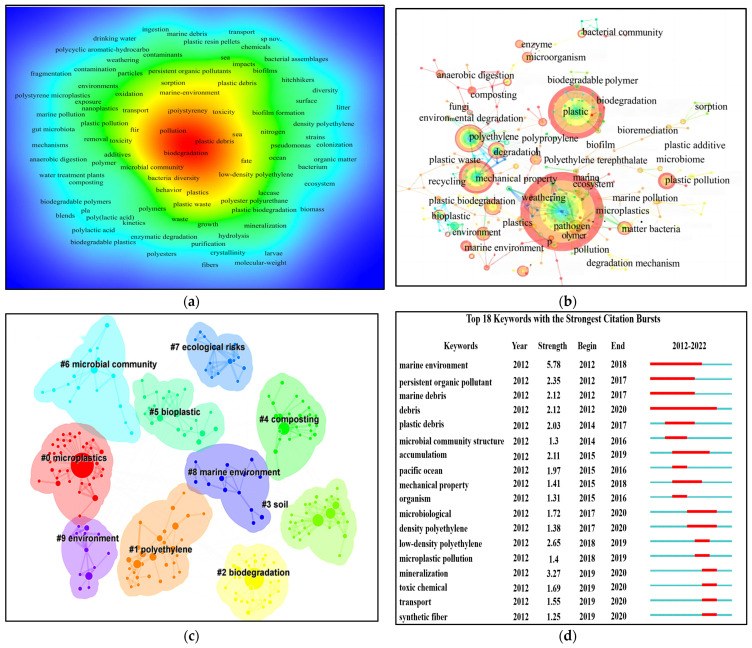
Keyword analysis: (**a**) visual map of the common occurrence density of keywords in the research on biological governance and mechanisms of microplastics from 2012–2022, the color of the annual cycle reflects the periods before and after the appearance of each keyword, as the color changes from purple to red, the period in which each keyword appeared becomes more recent, and vice versa; (**b**) network visualization map of co-occurrence keywords from 2012–2022, the color of the annual cycle reflects the periods before and after the appearance of each keyword, as the color changes from purple to red, the period in which each keyword appeared becomes more recent, and vice versa; (**c**) VOSvileviewer network diagram of research on biological governance and mechanisms of microplastics from 2012 to 2022, each node denotes a keyword and the keywords are clustered with different colours; (**d**) keywords with the strongest citation bursts, the red bars represent frequently cited keywords, while the green bars were infrequently cited keywords.

Keywords are words that have substantial meaning in expressing the central content of a paper and highlighting the key research directions in a field [36]. The keywords from 331 microplastic studies were summarized and counted. A density visualization of the chromatography based on keywords and hotspot intensity is displayed in Figure 4a, with warm and cold colors representing hot and cold spots, respectively. Microplastics, biodegradation, degradation, polyethylene, the microbial community, and microorganisms are common keywords with the highest density.

The color of the annual cycle reflects the periods before and after the appearance of each keyword. As the color changes from purple to red, the period in which each keyword appeared becomes more recent, and vice versa. By analyzing the key research directions from 2012 to 2022 (Figure 4b), it is identified that microplastic biodegradation and mechanism research has received extensive attention in the past five years, especially after 2019. This indicates the urgency of the microplastic pollution problem that needs to be solved and the new possibilities offered to this field with the development of novel biotechnologies.

The network diagram of research on the biological governance and mechanisms of microplastics for 2012–2022 (Figure 4c) illustrates the multiple topics involved in this field. There are nine clusters of keywords, in order from 0 to 8; the smaller the number, the more keywords there are. Each cluster is a composition of multiple closely related words, each with a different color. The main studies have focused on microplastics (7.87, 0.01); biodegradation (6.59, 0.05); marine pollution (5.15, 0.05); ecosystem services (5.15, 0.05); and fragmentation (5.15, 0.05).

Figure 4d facilitates the identification of the hot topics. The red lines in the diagram are heat bars, representing the time period of the strongest citation bursts [37]. The first keyword related to the marine environment was found in 2013. Biodegradation was a hot topic from 2013 to 2018, and has been the focus of research since 2017. Based on the above studies, we show that there are many studies on the microbial degradation of microplastics in the marine environment compared with the atmospheric and soil environments. The current degradation of bacteria and the studied degradation mechanisms are all found in the marine environment. However, the microplastic content in the atmospheric and soil environments cannot be underestimated, and understanding its biodegradation process and mechanism is very essential.

## 4. Discussion

### 4.1. The Status of Microplastic Biodegradation Research

A total of 331 articles were published from 2012 to 2022. Since 2012, the number of papers published has shown a trend of continuous growth. This indicates that the study of the biological governance and mechanisms of microplastics has developed rapidly during this period. The number of papers published in the past two years (2021–2022) is 62 more than in the previous decade. This may be due to the widespread concern about the dangers posed by microplastics. Microplastics have been shown to exist in plants and animals, entering through the human body along with the food chain, and in the human body through the blood circulation into multiple organs. It has been shown that when white blood cells in the human body swallow a certain amount of microplastics, they will die, releasing enzymes and causing inflammation. This then attacks more white blood cells, triggering continuous activation and leading to a variety of inflammatory and respiratory illnesses [38]. Therefore, a series of policies on microplastic control have been issued by various countries, such as the 2019 United Nations (UN) report, “Single-Use Plastics and Microplastic Legal Restrictions”, “The Toxic Substances Control Act” of 2020, and “The New Pollutants Management Action Program” enacted by China in 2021, which provide direction and support for microplastic pollution management.

The top three countries in which articles were published were China, the United States of America, and India. Estonia, the Maldives, Costa Rica, and other countries had only one article. This indicates that China is the largest contributor to microplastic biodegradation research among developing countries.

In terms of the authors’ countries, the top 10 authors are all Chinese. This shows that microplastic pollution is a hot topic of a lot of Chinese researchers in the field. However, we found that 112 authors (60.86% of the total authors) had published only one paper. This indicates that microplastic biodegradation is an emerging field, and although there are many researchers, there are still few results are still few and there is a lot of room for development.

The United States of America was the country that worked the closest with China. Kenya, Montenegro, Uganda, and other countries rarely collaborate with foreign countries. The process and mechanisms of microplastic biodegradation are a comprehensive field covering disciplines such as macrogenomics, enzymology, and chromatographic analysis. It is necessary to strengthen domestic and international cooperation in order to achieve further development in this field.

### 4.2. The Current Status of Functional Microorganism Development for Microplastic Biodegradation

**Figure 5:**
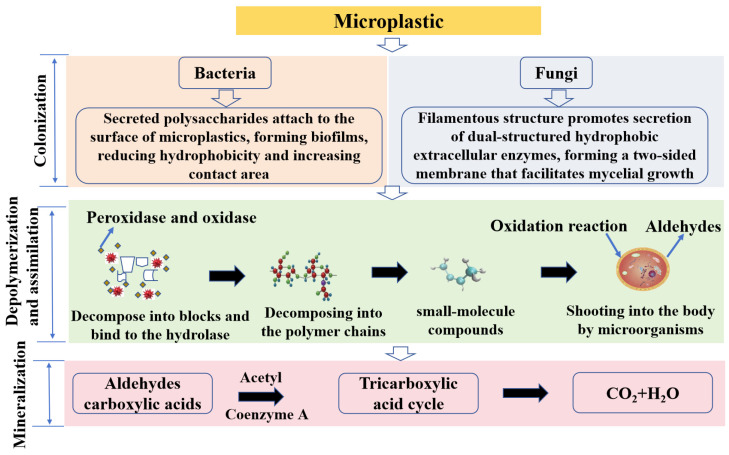
Microplastic biodegradation processes.

Microplastics can provide a habitat for many special microbial communities, such as microplastic-degrading bacteria, that can change the ecological function of the ecosystem and play a role in microplastic degradation [39]. Microplastics are polymeric substances that have existed in the environment for a long time and do not degrade easily [40], remaining in the environment for a decade or longer. It has been found that some microorganisms in the ecosystem have significant degradation effects on microplastics. This includes bacteria and fungi, which can participate in the degradation of microplastics [28]. However, it is difficult for a single strain to completely degrade microplastics, and complexes of several types of bacteria are needed instead to be effective for the degradation of microplastics [20]. In addition to being widely distributed on microplastic surfaces, these functional microorganisms are also present in the digestive tract of some animals [41]. Whether it occurs through bacteria, fungi, or intestinal microorganisms, the main principle of microplastic biodegradation is a complete decomposition of microplastics into harmless inorganic substances through microbial metabolism. Microplastic biodegradation generally consists of three steps: First, microorganisms colonize the surface of the microplastic and form small particles. Second, cleavage occurs under the action of enzymes to form small molecular compounds. Third, the microplastics undergo mineralization and decomposition into CO_2_ and H_2_O. Bacteria have faster growth and reproduction, strong genetic manipulation, and clear metabolic pathways, which make it easier to elucidate the degradation process and mechanism in studies. Fungi have a higher degradation efficiency and strategy, their enzyme system is powerful, and their filamentous structure facilitates the secretion of extracellular enzymes, which can act on microplastics, enhance adsorption capacity, and generate biosurfactants; these characteristics are lacking in bacteria (Figure 5) [42].

With the development of biotechnology, high-throughput sequencing technology has been widely applied to the study of microbial functional genes, which can be used to precisely probe the molecular mechanisms underlying certain functions. Firstly, it uses whole genome sequencing to obtain all the genetic information about degrading bacteria, and then the data are annotated and analyzed to see if the strains have gene-encoding enzymes with microplastic degradation potential and metabolic genes of microplastic metabolism-related pathways. Moreover, through genetic engineering techniques such as gene mutation, the degradation enzyme genes can be targeted for mutation to improve the degradation activity of the enzyme, and enzyme preparations can be obtained in bulk through mass replication in the strains. Finally, the degradation mechanisms of microplastics were investigated using genomics, secreted proteomics, and metabolomics.

#### 4.2.1. Identified Microplastic Degradation of Bacteria and Degradation Mechanisms

Bacteria are one of the main groups of organisms and have the largest number of species of all organisms. There have been many reports on the bacterial degradation of microplastics. Plate separation is primarily used to isolate and purify degradative bacteria from samples in order to obtain single strains capable of degradation. Table 3A summarizes the degraded bacterial taxa reported to date. The selected microplastic-degrading bacteria belonged to four phyla, among which the families γ-Proteobacteria and Actinobacteria were the largest.

As more bacteria are found to reduce microplastic content, their degradation properties and effects on microplastics are receiving increased attention. Firstly, microplastics are eroded under the action of enzymes, oxidizing and weakening the structure, introducing polar groups into the hydrocarbon skeleton, and promoting a stronger attachment of microorganisms. The formation of small aliphatic hydrocarbons increases the possibility of bacterial uptake, and through several steps of dehydrogenation and C-C bond breaking, bacteria finally enter the tricarboxylic acid cycle, which provides the required carbon source and energy for bacterial metabolism.

The bacterial degradation of microplastics is a popular method. With the advancement of research, it has been found that the microplastic weight loss rate is low when bacteria are used for degradation, approximately 0–15%. In addition, the use of bacteria to degrade microplastics is a relatively long process, approximately 0–3 months. Therefore, shortening the degradation process of microplastics, improving the degradation rate of bacterial microorganisms, optimizing culture conditions, and developing improved degradation strains will be the focus of research in the future.

#### 4.2.2. Identified Microplastic Degradation and Degradation Mechanisms in Fungi

Fungi are a large group, and their filamentous structure promotes the secretion of extracellular enzymes that can enable them to be better adapted to different environments for polymer degradation, such as dissolved organics [43,44]. Fungi seem to degrade polyethylene at a higher rate compared to bacteria [45]. As shown in Table 3A, the fungi that can degrade microplastics mainly belong to Ascomycota and Aspergillus, including *Aspergillus*, *Penicillium*, and *Rhizopus*.

Microplastic degradation by fungi involves the secretion of extracellular enzymes in the presence of filamentous structures. The dual structure of the hydrophobic protein produced by fungi acts on the surface of microplastics to form an amphipathic film that promotes the attachment of mycelium [46], which decreases the structural properties of microplastics [47] and depolymerizes to form oligomers and monomers [48]. Through a membrane transport system, these small molecules pass through a semipermeable membrane and are assimilated by the fungus [49].

Fungi have a high degradation efficiency, and their use in microplastic degradation is becoming a new research trend. The next step is to focus more efforts on screening and studying functional fungi that can degrade refractory microplastics. This will provide theoretical and technical support for the biological governance of the environment, aiming to reduce the impact of microplastics [28,50].

#### 4.2.3. Microplastic Degradation by Insect Gut Microorganisms and Its Mechanism

Some insects can chew and eat beeswax or plastic products and use them as their sole carbon source, providing a powerful biological resource for the biodegradation of microplastics [51]. Eight insect species have been reported to feed on and degrade plastic, and most studies have focused on yellow mealworms, barley worms, and wax borer larvae (Table 3B). Larval-stage yellow mealworms, earthworms, and wax borers swallow and, with the help of the microorganisms in their bodies, degrade various plastic polymers [52]. The microorganisms in these insects’ bodies enable them to degrade microplastics. Therefore, in addition to isolating biodegradable microplastics directly from plastic rings, screening for microplastic-degrading bacteria in these insects has become a viable option.

#### 4.2.4. Microplastic Degradation Enzymes

Fungi and bacteria are able to degrade microplastics through the secretion of various degrading enzymes. Many scholars have begun to degrade microplastics through direct enzyme synthesis. Microplastic-degradative enzymes can be classified as extracellular enzymes and intracellular enzymes according to their position. Microplastic enzymes involved in long-carbon-chain separation, which make plastic polymers form oligomers or dimers, can be absorbed into the cell. However, since research on microplastic-degrading enzymes is fairly new, few studies have investigated the enzymes during the microbial degradation process, and the focus has mainly been on extracellular enzymes. Currently, the enzymes found to play important roles in the degradation process of microplastics are proteases, lipase, keratinase, laccase, manganese peroxidase, lignin peroxidase, and alkane hydroxylase [53,54]. Polymers can undergo enzymatic biodegradation. However, for the enzymatic degradation of microplastics, five important factors must be addressed: surface topography, crystallinity, water absorption, polymer chain orientation, and reaction temperature. Studies have been reported on the application of polyethylene terephthalate hydrolases in fibers, films, and nanoparticles. The enzymatic hydrolysis of other microplastics has also been the target of previous studies that focused on the enzymatic surface modification of polymer fibers (changing the functional groups on the surface of microplastic fibers) and the treatment of microplastic waste (degradation of fiber building blocks). In other words, surface modification improves the wettability, fastness, dyeing, and pilling resistance of microplastics and ultimately the hydrophilicity of plastics. The surface area-to-volume ratio plays an important role in the degradation process; the higher or lower this ratio is, the more easily the polymer can degrade compared to fiber or film [55,56,57].

### 4.3. Current Status of Development of Biodegradation of Different Types of Microplastics

Polyethylene (PE), Polystyrene (PS), and Polyethylene Terephthalate (PET) are common plastic materials that are widely used in daily life. Microplastics formed after crushing are degraded under the action of microorganisms, and the degradation processes and mechanisms of different microplastics differ significantly due to their different compositions. In addition, the degradation process and mechanism will also change under the influence of environmental factors in actual degradation. PE microplastics are straight-chain alkanes with a high degree of polymerization and strong flexibility. Their degradation process consists of two stages: the first is the depolymerization process under the action of peroxidase, which depolymerizes the polymer into oligomers with 10–50 C atoms; the second is the mineralization stage after hydroxylation, which achieves the degradation of PE microplastics through the tricarboxylic acid cycle. PS microplastics are aromatic polymers that degrade under the action of a series of enzyme-catalyzed reactions such as dehydrogenation, deoxygenation, and hydroxylation. Microbial degradation is accomplished through two different metabolic pathways, namely, the styrene-ethyleneglycol-3-ethylene diphenol pathway and the styrene oxides-phenylacetaldehyde pathway. The biodegradation of PET microplastics starts with the PETase enzyme, which generates ethylene glycol (EG), monohydroxyethyl tetrahydronaphthalene (MHET), and Bis(2-hydroxyethyl) terephthalate (BHET), and then moves to degradation by terephthalic acid (TPA), ethylene glycol (EG), and ultimately the tricarboxylic acid cycle (TCA) through oxidase, which completes the biodegradation of PET microplastics [58].

**Table 3:** (**A**) Typical bacteria and fungi with microplastic degradation functions. (**B**) Microplastic-degrading insects and their internal microorganisms involved in degradation.

**(A)**					
**Taxon**	**Isolate**	**Time**	**% of Degradation**	**Microplastic**	**Reference**
Bacteria-Proteobacteria	*Acinetobacter pitti IRN19*	4 w	26.8 ± 3.04	LDPE	[59,60]
	*Pseudomonas*	8 w	5.95 ± 0.03	PE	
	*Alcanivorax borkumensis*	80 d	3.5 ± 0.34	LDPE	
	*Pseudomona saeruginosa*	60 d	51	LDPE	
	*Achromobacter*	150 d	9	HDPE	
	*Comamonas*	90 d	46.7	PE	
	*Enterobacter*	30 d	12	HDPE	
	*Microbulbifer*	90 d	36.88 ± 1.25	HDPE	
	*Stenotrophomonas*	90 d	18.18 ± 0.69	HDPE	
Bacteria-Firmicutes	Geobacillus stearothermophilus FAFU011	56 d	4.20	PS	
	Paenibacillus sp.	60 d	14.7	PE	
	*Lysinibacillus*	90 d	36.88 ± 1.25	HDPE	
Bacteria-Actinobacteria	*Rhodococcus* sp. *IR-SGS-T6*	60 d	1.58	LDPE	
	*Nocardia* sp. *IR-SGS-T3*	60 d	1.58	LDPE	
	*Streptomyces* sp.	60 d	1.58	LDPE	
	*Kocuria*	60 d	14.7	PE	
Bacteria-Bacteroidetes	*Sphingobacterium*	28 d	3.04	LDPE	
Fungi-Ascomycota	*Aspergillus* sp.	30 d	-	PP/PBAT	
	*Fusarium* spp.	28 d	30	LDPE	
	*Cladosporium pseudocladosporioides T1.PL.1*	14 d	65	PU	
	*Penicillium* sp.	30 d	-	PP/PBAT	
	*Trichoderma*	28 d	-	PE	
	*Alternaria*	120 d	52.02	PE	
	*Lasiodiplodia*	90 d	-	LDPE	
**(B)**					
**Insect Name**	**Microplastic**	**Degradation Effect**		**Function Microbial**	
Waxworms, Indian Mealmoths	Polyethene	Chew and feed on polyethylene; microbial degradation in the body		*Nitrobacteria asburiae YT1*, *Bacillus* sp. *YPI*	
Zophobas Morio	Polystyrene	Using polystyrene as the carbon source, the weight and number in feces are reduced, and microbial degradation in vivo		*Klebsiella*, *Citrobacter*	
Mealworms	Polyethene, polystyrene	Using these two microplastics as carbon sources, there is microbial degradation in the intestine with a combined effect		*Citrobacter* sp., *Kosakonia* sp.	
Dark Mealworms	Polystyrene	Using polystyrene as a carbon source, polystyrene depolymerization was found in the gut		*Enterobacteriaceae*	
Waxworms	Polyethene	Microbial degradation bacteria were identified in the gut		*Enterobacter* sp. *DI*	
Yellow Mealworms	Polystyrene	Microbial degradation bacteria were identified in the gut and feces		*Aspergillus niger KHJ-1*	
Earthworm	Low-density polyethylene	Microbial degradation bacteria were identified in the gut		*Rhodococcus jostii*, *Mycobacterium*	

## 5. Conclusions

This review employs bibliometric analysis to select articles on microplastic biodegradation and mechanisms from 2012 to 2022 and systematically summarizes the current research hotspots and progress in microplastic biodegradation and its mechanisms through the study of annual publication volume, countries, institutions, authors, and keywords. Based on the results, 2018 to 2022 was the rapid growth phase of publications on microplastic biodegradation. In this period, 81 countries and 308 institutions started research in this field, with significant contributions from Chinese scholars and research institutions. According to the statistics, China’s research volume accounted for 32.7%, the highest percentage. Secondly, the top three authors and institutions are from China, of which the Chinese Academy of Sciences represents 8% of the publications, works closely with other institutions at in China and abroad, and is the most active in academic exchanges. Zhang Yong is the author of the largest volume of publications and Li X is an author with whom many authors have cooperated closely. However, at present, most of these authors are concentrated in China, and communication with foreign scholars needs to be strengthened. From the keyword network diagram, microplastics, biodegradation, and the marine environment have been research hotspots in recent years. Although we have carried out a great deal of related research, problems are still encountered in biodegradation, such as unknown processes and unclear mechanisms, which need to be solved urgently. Based on the current research progress, the following aspects should be focused on in the future:

1. Only a few types of degrading strains have been identified. Fungi are currently only found to degrade polyvinyl chloride (PVC). The role of bacteria and enzymes needs to be investigated further.
2. Holistic knowledge of the degradation process needs to be obtained. Current research on the biodegradation and mechanisms of microplastics mainly focuses on the marine environment, with less data available for the soil, water, and atmospheric environments. This limits a holistic understanding of microplastic degradation and makes describing its mechanisms more challenging. Therefore, there is a need to investigate the processes by which microplastic degradation occurs in different environments and the links between them.

## Figures and Tables

**Table 1:** The top 10 authors and co-cited authors of microplastics research (N%).

Rank	Author	Country	N (%)	Co-Cited Author	Country	Citations
1	Zhang Y	China	17 (3.27%)	Lin Y	China	356
2	Li Y	China	12 (2.31%)	Li C	China	264
3	Wang J	China	12 (2.31%)	Li X	China	212
4	Chen J	China	9 (1.73%)	Li C	China	157
5	Chen Y	China	8 (1.54%)	Li X	China	119
6	Sun Y	China	8 (1.54%)	Li X	China	112
7	Zhang S	China	8 (1.54%)	Zhang S	China	76
8	Zhang Z	China	8 (1.54%)	Xie Y	China	72
9	Liu Y	China	7 (1.35%)	Lin Y	China	172
10	Wang X	China	7 (1.35%)	Li C	China	85

**Table 2:** Top five co-cited journals.

Rank	Cited Journals	Count	Year
1	Environmental Science & Technology	280	2012
2	Science of the Total Environment	267	2016
3	Environmental Pollution	263	2012
4	Marine Pollution Bulletin	241	2012
5	Chemosphere	234	2012

## Data Availability

Data will be made available on request.

## Abbreviations

ACS	American Chemical Society
ASM	American Society for Microbiology
UCAS	Chinese Academy of Sciences
NWAFU	Northwest A&F University
CAAS	Chinese Academy of Agricultural Sciences
USA	The United States of America
PAEs	Phthalates
PBDEs	Polybrominated Diphenyl Ethers
BPA	Biphenyl A
POPs	Persistent Organic Pollutants
PLA	Polylactic Acid
PBAT	Polybutylene Terephthalate
PCL	Polycaprolactone
PS	Polystyrene
BP	Bacillus parapsilosis
BC	Bacillus cereus
PP	Polypropylene
PE	Polyethylene
UN	United Nations
PET	Polyethylene Terephthalate
EG	Ethylene Glycol
MHET	Monohydroxyethyl Tetrahydronaphthalene
BHET	Bis(2-hydroxyethyl) terephthalate
TPA	Terephthalic Acid
TCA	Ultimately The Tricarboxylic Acid Cycle
LDPE	Low Density Polyethylene
HDPE	High Density Polyethylene
PU	Polyurethane
